# A qualitative study on the health system-related needs in women survivors of rape

**DOI:** 10.1186/s12913-024-10852-0

**Published:** 2024-04-09

**Authors:** Leila Asadi, Mahnaz Noroozi, Hajar Salimi, Sara Jambarsang, Fardin Mardani

**Affiliations:** 1 Student Research Committee, School of Nursing and Midwifery, Isfahan University of Medical Sciences, Isfahan, Iran; 2 Research Center for Nursing and Midwifery Care, Shahid Sadoughi University of Medical Sciences, Yazd, Iran; 3 Department of Midwifery and Reproductive Health, School of Nursing and Midwifery, Isfahan University of Medical Sciences, Isfahan, Iran; 4 Behavioral Sciences Research Center, Department of Psychiatry, Isfahan University of Medical Sciences, Isfahan, Iran; 5 Research Center of Prevention and Epidemiology of Non-Communicable Disease, Department of Biostatistics and Epidemiology, School of Public Health, Shahid Sadoughi University of Medical Sciences, Yazd, Iran; 6 Forensic Medicine Research Center, Isfahan, Iran

**Keywords:** Reproductive health services, Survivors, Rape, Woman, Qualitative research

## Abstract

**Background:**

Rape, as an adverse incidence, leads to irreparable complications and consequences in women. Provision of health services to women survivors of rape requires catering for their real needs and identifying current deficits as well as barriers. The present study aimed to explore health system-related needs in women survivors of rape.

**Methods:**

In the present qualitative study, the participants consisted of 39 individuals, including 19 women survivors of rape and 20 individuals with work experience in providing services to women survivors of rape. The participants were selected using the purposive sampling method with a maximum variation in Isfahan, Iran. Data were collected through in-depth interviews as well as field notes and were concurrently analyzed via conventional qualitative content analysis method.

**Results:**

After analyzing the interviews, the health system-related needs of women survivors of rape were classified into two main categories: 1- The need for efficient medical care services with three sub-categories, namely “receiving services with respect for privacy and confidentiality”, “non-judgmental behavior and approach”, and “the need to receive empathy and the feeling of not being alone”, and 2- The need for desirable conditions and structure to provide services with two sub-categories, namely “the need to receive comprehensive and integrated services”, and “establishing specialized centers for providing services to survivors”.

**Conclusions:**

Overall, explaining and highlighting the health system-related needs of women survivors of rape could provide a suitable basis for policy-making and planning according to their real needs. Receiving continuous services in separate centers with confidentiality and empathy could reduce the worries and concerns of women survivors of rape and help improve their health.

**Supplementary Information:**

The online version contains supplementary material available at 10.1186/s12913-024-10852-0.

## Background

Rape, as a violation of health and human rights, affects numerous women worldwide [1]. The prevalence of rape is different in every region in the world. Dworkin et al., who studied the prevalence of rape based on studies in the last 10 years, reported a mean rape prevalence of 29% in women in different countries, and 59% in some countries, including South Africa, indicating a very high prevalence of rape in this region [2]. Statistics related to rape in many countries are not accurate for various reasons such as lack of reporting by survivors. It is believed that about 80% of rape cases go unreported for various reasons [3, 4]. In Iran, as with many countries in the world, due to the taboo and fear of social stigma, there are no accurate and reliable statistics on rape cases. Nevertheless, the lack of statistics is not a reason for the absence of such a problem. In some studies, incest rates are reported to be about 22–25% [5].

Women survivors of rape experience a large number of short and long-term consequences in different aspects of health [6]. The most important psychological consequences include post-traumatic stress disorder (PTSD), as well as other anxiety disorders, depression, and suicide [7–11]. Other implications include sexually transmitted infections (STIs), HIV/AIDS, chronic diseases, physical injuries to the body and genital area, unwanted pregnancy, and unsafe abortion [12–15].

Even though rape is a very important threat to the health of many women, the use of health services is very low among survivors in many societies [16, 17]. The provision of health services to survivors of gender-based violence, including rape, has been significantly developed since 2000 thanks to the extensive activity of reproductive health activists. Based on a global evaluation of sexual and reproductive health in 2004, the clinical management of rape in humanitarian settings is associated with many deficits, but such services were more widely available 10 years later [18]. Despite the considerable developments, the provision of suitable services to survivors faces problems owing to several reasons, including insufficient budgets of countries, lack of correct referral of survivors, limited supply and stock of drugs, and gender biases in societies [19, 20]. The survivors also seldom use these services even in countries where such barriers have been removed [19]. Women survivors of rape face numerous barriers to receiving health services, including stigma, rejection, and non-confidentiality, leading to under usage of health services by survivors [20]. Meanwhile, the needs of women survivors of rape should be recognized and they should receive suitable services and support according to their needs. Providing services while neglecting their needs will cause them to be re-victimized and suffer more injuries [21].

Knowledge about perceptions and experiences of women survivors of rape regarding services, as well as the existing barriers and deficits in healthcare systems is significantly important in comprehensive and cultural-based interventions for improving health services, and ultimately, their health. There is little research on the proper and desirable services of this group of women, and it is not yet clear whether the existing services are fully tailored to their needs. Qualitative research is an approach to discovering and describing the experiences of participants and conceptualizing them. It could enhance insight, perception, and knowledge of human experiences [22]. Considering the differences in the social and cultural context of Iran with other countries as well as the importance of sexual and reproductive health of women survivors of rape, this qualitative study aimed to explore the health system-related needs of women survivors of rape.

## Methods

This research was part of a large mixed-methods study conducted with a content analysis approach from November 2021 to August 2022.

### Settings, sample, and recruitment

The participants of the study included women survivors of rape (*n* = 19) and individuals with work experience in providing services to them (*n* = 20) in Isfahan, Iran. The selection of rape survivors began through purposive sampling and then continued with a strategy of maximum variation regarding age, job, education level, marital status, number of pregnancies and deliveries, and length of time after rape. Subsequently, a number of participants were included in the research using the snowball method based on the introduction of the primary participants. The inclusion criteria for women survivors of rape were informed consent and willingness to participate in the interview, being able to communicate and express experiences of rape, Iranian nationality (due to the difference in the needs of rape survivors according to cultural and social conditions and background), being 21 years old or older during the research [23], a minimum of 6 months and a maximum of 2 years past the rape [10, 24], lack of well-known psychological disorders and a history of psychiatric treatment before rape or now (based on the participant’s report), no previous experience of rape, and no experience of adverse incidents in life (death of a family member, etc.) over the past three months. Exclusion criteria included reluctance to continue participation in the study at any stage of the study. Service providers were also first selected via purposive sampling, and then sampling continued with a strategy of maximum variation in terms of work experience. Inclusion criteria for service providers included willingness to participate in the study plus having expertise and work experience in the field of rape.

In the present study, access to the participants was done through the special counseling centers for vulnerable women, Drop in Centers (DIC), addiction treatment centers, social emergency, together with women’s rehabilitation centers, midwives and reproductive health specialists’ offices, counseling and midwifery service centers, emergency and women’s and midwifery clinics of hospitals, legal medicine centers, counseling centers for behavioral diseases, private offices of general practitioners and specialists (obstetrics and gynecology, infectious diseases and psychiatry), psychology and counseling centers, guidance and counseling centers at universities, social deputy of the Law Enforcement Force, and Crime Prevention Unit (affiliated to the judicial system) of Isfahan city. In these centers, participants were invited to the study through phone calls. In order to maintain confidentiality, phone calls were made to the women survivors of rape and the criteria for their entry into the research were checked by the service providers working in the centers; the researcher was able to access their contact information only after obtaining informed consent from the survivors. In the present study, no one refused to participate or dropped out of this study once they were recruited to participate. The first author (L.A) had no previous relationship with the participants and centers.

### Data collection

In the present study, data collection methods included semi-structured in-depth interviews and field notes. The first author (L.A) conducted the interviews and filed notes. She had 11 years of working experience in midwifery and was a Ph.D. candidate in reproductive health in Isfahan University of Medical Sciences. Four other authors had previous experience in qualitative paper/report writing and interviewing. Prior to data collection, the first author (L.A) wrote down initial preconceptions about the study topic based on her previous working experience and from literature review. Questions, prompts, and guides were provided and this was piloted in two pilot interviews. Interviews with women survivors of rape began with the general questions: *“What problems have you faced in receiving health services since this incident (rape)? What needs did you feel in this regard? Please explain it.“*, and then the participants’ open and interpretative answers guided the process. The interviews with service providers began with the general question: “*What do you think women, who have experienced rape, need about the healthcare system? Please explain it.“*, and then the participants’ open and interpretative answers guided the process. All interviews were digitally recorded using an MP4 device. In this research, 39 interviews (lasting 45 to 60 min) were conducted in participants’ preferred places (such as parks). No one else was present at the interview besides the participants and the researchers. The interviews continued until data saturation was reached by interviewing 36 persons, with no new code being formed, and all codes previously obtained and duplicated. However, to avoid false data saturation, the researchers conducted another three interviews after repletion of codes in interview NO.36, to be more confident of achieving accurate data saturation, with no new data in the next three interviews. At this point, the researchers concluded that they would stop the data collection and analysis since data saturation had been obtained. In the present study, the first author (L.A) recorded observations of participants’ non-verbal behavior and interaction in field notes.

### Data analysis

In the present study, conventional qualitative content analysis based on the method by Graneheim and Lundman was used to analyze the data [25]. Data analysis was performed manually where no software was used. The data were regularly transcribed after each interview. Then, the interviews were read repeatedly to obtain a complete understanding of them. Sentences and expressions were coded by the first author (L.A) and after the formation of codes by the inductive method, similar codes were merged; those with similar meanings were placed in one category and created sub-categories. Thereafter, the conceptually similar categories were placed in a main category and constituted the main categories.

### Rigor and trustworthiness

A variety of methods, including in-depth interviews at different times and places, field notes, and selecting participants with a maximum variation were utilized to validate the results. In the present study, the results were presented to three women survivors of rape with the same characteristics as the participants, who did not participate in the study, to judge the similarity of the study results with their own experiences so that the transferability could improve. To enhance the confirmability of the data, coded interviews were discussed with five participants in other meetings and their final comments were summarized to achieve a review by participants. To augment the dependability of the results, the opinions of five experts were used to match and ensure the consistency of the results with the participants’ statements.

### Ethical considerations

The research was approved by the ethics committee of Isfahan University of Medical Sciences (approval code: IR.MUI.NUREMA.REC.1400.133). Informed consent, right to withdraw at any time, confidentiality, and preservation of anonymity were respected in this research. The objectives for the study were explained prior to each individual interview.

## Results

The demographic characteristics of the participants are reported in Table 1. A total of 34 inferential codes, five sub-categories, and two main categories were extracted after data analysis. The two main categories included “The need for efficient medical care services” and “The need for desirable conditions and structure to provide services” (Table 2).

**Table 1:** Demographic characteristics of participants

Women survivors of rape (*n* = 19)	Age (years)	21–45
	Time after rape (months)	6–20
	Education	High school (1), diploma (7), B.S. (10), M.S. (1)
	Occupation	Housewife (6), Employee (3), Service job (2), Unemployed (6), Self-employed (2)
	Marital status	Single (13), Married (6)
	The relationship between the survivor and the offender	Acquaintance (13), stranger (6)
Service providers (*n* = 20)	Age (years)	26–56
	Work experience (years)	2–29
	Expertise	Obstetricians (2), Reproductive health specialist (2), Midwife (3), Lawyer (2), Emergency medicine specialist (1), Infectious diseases specialist (1), Psychiatrist (3), Psychologist (2), Social worker (1), Forensic medicine specialist (2), Physician (1)

**Table 2:** Results of data analysis

Code	Sub -category	Main category
Fear of revealing the name when going to the midwife	Receiving services with respect for privacy and confidentiality	The need for efficient medical care services
The need to receive OCPs from the pharmacy anonymously		
Concern about non-observance of confidentiality by the consultant		
The need to receive services anonymously at the health center		
The need not to be judged by health care providers	Non-judgmental behavior and approach	
The need to not be judged when getting OCPs from the pharmacy		
Not going to doctors and midwives because of the fear of being judge		
The need to receive empathy from the counselor	The need to receive empathy and the feeling of not being alone	
The need to feel that she is not the only person who has experienced this event		
The need for a chain of service delivery	The need to receive comprehensive and integrated services	The need for desirable conditions and structure to provide services
The need for a center to receive psychological services along with receiving other needed services		
The need to receive all services comprehensively and in one center		
Feeling uncomfortable with receiving services among other clients	Establishing specialized centers for providing services to survivors	
The need for separate and dedicated centers to receive all the required services		

### 1-The need for efficient medical care services

According to the participants, the health services provided to the survivors were inefficient and did not meet their needs. Many of the participating survivors expressed their discomfort with the lack of respect for privacy and confidentiality while receiving services, recording personal information when providing services, wrong behaviors and judgments, and lack of understanding, acceptance, and empathy. Further, many of these women faced such issues when going to the centers, then left the center or gave up receiving health services. This main category was composed of three sub-categories, namely “receiving services with respect for privacy and confidentiality”, “non-judgmental behavior and approach”, and “the need to receive empathy and the feeling of not being alone”.

#### A-Receiving services with respect for privacy and confidentiality

Participating rape survivors were worried about the disclosure of their names and identity when visiting healthcare centers. They expressed that they needed special attention from service providers to maintain confidentiality and non-disclosure of their identity information at all stages. For this reason, many of them had refused to go to pharmacies and health centers owing to being worried about their identity information being revealed.

*“I was frightened to go to the doctor. I was afraid that someone might know my name. For example, I used to say (to myself) that they would know my name and recognize me, lest they wrote my name in a list.” (A 37-year-old survivor)*.

*“… I was worried that if I go to the pharmacy to buy pills or go to the doctor, my information would be read and they would record what medicine I took.” (A 35-year-old survivor)*.

The service providers narrated that most survivors of rape were worried about the disclosure of their identity when they were in healthcare centers, and thus they often refused to continue attending those centers and received incomplete services.

*“… When the result of their HIV or HBS Ag tests are positive and we inform them, either they don’t come to receive medication and continue treatment and we have to follow up regularly, or they insist on not recording their test results in their files.” (Midwife)*.

#### B-Non-judgmental behavior and approach

According to most participating survivors, when they received healthcare services, they face service providers’ judgmental behavior which was very annoying for them. They were dissatisfied with the fact that they were considered guilty in the incident of rape and the behavior of health service providers that made them feel guilty. Therefore, they did not express real health problems and needs due to the worry and embarrassment of being judged.

*“… When I went to the pharmacy and took pills, that man looked at me in an unsatisfactory way. In these cases, society looks at girls very badly. They always think that it is often due to girls’ fault. “(A 37-year-old survivor)*.

*“… When I went to the laboratory, they didn’t treat me well. They never talked to me. I gave the doctor’s prescription to take a specimen from me. After reading, he looked at me and just said: Go sit in the room. His behavior was annoying.” (A 25-year-old survivor)*.

The service providers argued that behavior with judgment and blame could be barriers to receiving correct healthcare services in survivors. When survivors were faced with such blaming behavior combined with judgment, they would have doubts about disclosing the rape, leading them to postpone receiving healthcare services.

*“… I had clients who came with other complaints, including anxiety and stress. When they received empathy and non-judgment, they slowly started to reveal the rape and ask for help.” (Psychiatrist)*.

#### C-The need to receive empathy and the feeling of not being alone

The participating rape survivors narrated that they needed to receive empathy from service providers due to the bitter experience of rape. Many of them expressed that they needed service providers to understand their situation and empathize so that they felt they were not the only people who were raped. They needed to receive the necessary support resources to disclose the rape incidence in the healthcare system. Many of them were annoyed by the lack of empathy. An important negative experience of these women about not receiving empathy was during the virginity examination.

*“… Here (The Forensic Medicine Organization), they didn’t pay attention to my feelings, nor to my voice and cries, and said like a normal patient, go upstairs and sleep. I had never been examined. My hands and legs were shaking.” (A 21-year-old survivor)*.

The service providers argued that if rape survivors received empathy and support from service providers, they would attend diagnostic and treatment processes more favorably and make more efforts to improve their health status.

*“Our behavior in the first meetings definitely has a great effect on not abandoning the person. When a person finds out we understand her and that she is not alone, it will definitely facilitate her future visits. In these women, not leaving care and follow-up is very important, where understanding and empathy with the surviving person should be a high priority for us.” (Psychiatrist)*.

### 2-The need for desirable conditions and structure to provide services

According to the results, healthcare services must be provided in optimal conditions and structures to meet the maximum needs of rape survivors. According to the participants, the current structure of health services did not fully cover the rape survivors’ needs and it required changes. This main category was obtained from the two sub-categories, namely “the need to receive comprehensive and integrated services”, and “establishing specialized centers for providing services to survivors”.

#### A-The need to receive comprehensive and integrated services

The participating rape survivors narrated that they were referred to different centers (including private clinics and offices, behavioral disease centers, medical centers, and centers affiliated with the welfare organization) to receive health services, and in each center, they received separate services that sometimes overlapped with other services. Based on the results, not only this issue led to spending a lot of time and money for the survivors, the lack of integrated services caused failure in receiving some essential services (e.g. diagnosis and treatment of STIs, contraception, supportive and psychological services). The impossibility of follow-up, failure to examine the survivors’ real needs at different stages after the rape, and providing interrupted services regardless of personal records were the most important disadvantages of such services.

*“… After my presence in the Forensic Medicine Organization, I went to the gynecologist’s office, and she gave me medicine for the infection. After some time, I still had a vaginal discharge. With my friend’s advice, I went to the infectious disease specialist and now I am taking a new medicine, but I still haven’t seen any difference.” (A 38-year-old survivor)*.

According to participating service providers, survivors of rape needed to receive comprehensive and integrated services provided by a team with different specialists comprising a gynecologist, a forensic medicine specialist, a psychiatrist, a social worker, and a psychologist to enhance their access to all necessary services and prevent long-term complications. They also recommended the establishment of centers for survivors of rape to provide services to them with long-term continuous follow-up.

*“The services and support for these women should be presented by a more specialized organization or group; hence, when these women visit, they know that the services are provided in a package, and all of their issues will be resolved without any problems.” (A forensic medicine specialist)*.

*“… The services for these women need to be presented by a team. Comprehensive, complete, and timely services should be provided, and this team should consist of gynecologists, social workers, psychologists, forensic medicine specialists, and psychiatrists.” (Psychiatrist)*.

#### B- establishing specialized centers for providing services to survivors

The lack of specialized centers to provide services to women survivors of rape is another deficiency of the healthcare systems which was taken into account and stated by the participating rape survivors.

*“… In my opinion, I wish the Forensic Medicine Organization would follow up on the issue. I wish they had a consultant here who would follow up on our bad situation and we could follow up on the spot because we don’t like to talk about this issue everywhere.” (A 23-year-old survivor)*.

The participating service providers argued that due to the rape survivors’ need to visit psychological, gynecological, midwifery, and laboratory centers to receive a variety of services, many of them did not present to these centers and missed necessary services due to the lack of special service centers. Also, they emphasized consideration of centers specialized in providing services to women survivors of rape.

*“… Most of the visiting survivors finally reach us after wandering around and visiting different centers and places.” (Gynecologist)*.

*“The (health) system should move towards a separate center for these women to receive necessary procedures and consultations so that when the examination is conducted (legally), this person knows that she does not need to go elsewhere to prevent pregnancy and infections.” (A forensic medicine specialist)*.

## Discussion

The present study aimed to explore the health system-related needs of women survivors of rape. The results indicated that women survivors of rape needed desirable, structured, and efficient care as well as treatment services. Survivors participating in the present study had experiences of non-preserved confidentiality, non-respected privacy, and blaming behaviors far from empathy in receiving healthcare services. There is a belief that women survivors of rape have many unmet physical and psychological needs making them dependent on health care systems. These needs should be identified and resolved in cultural contexts [26]. In this regard, the results of a systematic review by Bach et al. indicated that service providers’ insufficient knowledge about the way of empathizing with rape survivors without judgment and preserving confidentiality was an important barrier to survivors receiving healthcare services [27]. Conducting an exploratory descriptive qualitative study through interviews with survivors of rape, Sebaeng et al. reported that health service providers should be sensitive to problems experienced by women survivors of rape, not consider them as survivors of the crime scene, nor think of just collecting medical and legal evidence as their duties [28]. Polite et al. also confirmed that physicians and service providers not only must comply with medical and judicial guidelines and perform their therapeutic duties, but also should provide support and build trust through understanding, empathy, and non-judgment of the survivors [29]. Munala et al. reported that survivors of rape were concerned about the attitude of physicians and healthcare providers, normalization of rape, and blaming them [30]. Ferdowsian et al. found that profession was significantly associated with beliefs and attitudes about sexual violence and survivors. Law enforcement professionals were more likely than health professionals and lawyers to indicate that survivors should feel ashamed [31]. Thus, women survivors of rape need to receive survivor-based healthcare services which include non-judgmental and empathic behaviors. Health service providers should be supportive, not judge the survivors, and consider survivors’ dignity while providing services to bring aobut an efficient service procedure. It seems that the existing health services for women survivors of rape in Iran need detailed policies and plans based on women^,^ s needs, since the absence or incomplete health services can re-victimize and have many consequences for them. The results of the present study emphasized providing survivor-based medical care services for women survivors of rape since the way of providing services to survivors greatly contributed to the process of incident disclosure by survivors and their presence in the next meetings and follow-ups. Also, our findings suggest a need for interventions that adequately address potentially harmful beliefs as well as attitudes of some professionals serving women survivors of rape.

The present study also found that the necessary services for women survivors of rape should be comprehensive, integrated, and be provided by a team. Furthermore, these services should be provided in specialized centers that were specific to women survivors of rape. In this way, they would be assured of the provision of necessary and efficient services with long-term continuous follow-up. Sebaeng et al. study also indicated that survivors of rape needed to receive healthcare services through integrated specialized “Sexual Assault Response Teams (SART)” which provide all integrated services in teams by different specialists in separate centers to enhance the survivors’ access to all essential services and prevent long-term complications [28]. Shahali evaluated the healthcare providers’ experiences in dealing with survivors of rape and reported that two important barriers for survivors to receive health services included the lack of a single center for the provision of essential healthcare services to survivors of rape and the lack of organized guidelines for interdepartmental interaction [32]. Sepeng et al. in a study in Africa reported that most rape survivors received managed care for acute psychological health problems, injuries, pregnancy, STIs, and HIV; however, few survivors of rape received managed psychological healthcare during follow-up care sessions. They attributed the low attendance of survivors in psychological follow-up sessions to the lack of an integrated and continuous medical care system [33]. Ades et al. observed that even though patients with a history of sexual assault often had essential health needs, which continued long after the traumatic incident, most of the available services to survivors of rape only focused on acute care immediately after the violence, and a few clinics managed long-term medical outcomes and consequences of rape in a specialized trauma center [34]. Bougard et al. evaluated the quality of service provision in comprehensive multi-specialty centers for providing integrated rape services in South Africa and reported that the quality of service provision was satisfactory in these specialized centers, and survivors were highly satisfied with the available services as they received all services in a specialized center [35]. The results of the present study emphasized women survivors of rape need care and treatment services according to their basic needs in such a way that continuous services and long-term follow-up are provided with respect for confidentiality and privacy without judgment, stigma, and blame. Also, establishing specialized centers, which provide all required healthcare services to women survivors of rape, is particularly important in improving the quality of care for these women in Iran.

### Practical implications

Recognizing health system-related needs in women survivors of rape could facilitate evidence-based policymaking, planning, and making necessary changes in the health system. In addition, the results of the present study can help service providers understand, evaluate, and recognize the needs of women survivors of rape so that they can try to meet these needs and solve existing problems to improve women’s health. It is expected that the results of the present study will be utilized as a basis for conducting wider and more comprehensive studies about women survivors of rape and result in the identification of newer research domains. Future studies are suggested to design, conduct, and evaluate effective survivor-based health programs for women survivors of rape in Iran.

### Strengths and limitations

Through presenting an image of health system-related needs in women survivors of rape for the first time in Iran, the present study could contribute to designing the necessary interventions to improve health of these women. The most important limitation of the present study was the survivors’ shame or modesty to talk about the rape due to cultural and social taboos in Iran. Therefore, attempts were made to build the participants’ trust by providing explanations about the importance of the issue, committing the researchers to preserve confidentiality, as well as providing a safe and calm space for effective communication. Furthermore, researchers faced difficulties in obtaining permission to access participants in a number of research environments. In this sense, the researchers had to attend the centers that had issued sampling permits as well as reached the participants using the snowball sampling method.

## Conclusion

Based on the results of the present study, necessary measures should be taken to provide efficient medical care services to women survivors of rape and develop a desirable structure for providing health services in such a way that they can receive necessary services continuously in special centers with confidentiality and empathy. These results can be helpful for policy-making, planning, designing successful interventions, as well as providing basic and comprehensive care programs to improve women’s health.

### Electronic supplementary material

Below is the link to the electronic supplementary material.

Supplementary Material 1

## Data Availability

The datasets generated and/or analyzed during the current research are not publicly available as individual privacy could be compromised but are available from the corresponding author on reasonable request.

## Abbreviations

PTSD: Post-Traumatic Stress Disorder
STIs: Sexually Transmitted Infections
DIC: Drop-in Centers
