# Supplementary material for: A qualitative study on the health system-related needs in women survivors of rape
Source: BMC Health Serv Res. 2024 Apr 9;24:443. doi: 10.1186/s12913-024-10852-0 (PMC11005254; doi:10.1186/s12913-024-10852-0)
Supplement: Supplementary file 1 — Supplementary Material 1 [file 12913_2024_10852_MOESM1_ESM.docx]

**Additional files**

**Additional file 1:** Interview guide during the face-to-face interviews with women survivors of rape for the study conducted to explore the needs related to the health system from the perspective of women survivors of rape and service providers in Isfahan Town, Iran, 2021-2022 (See methods section for further description).

**Introduction:** *Aim, to create appropriate atmosphere*

- Name of the interviewer and affiliation
- Purpose of the study
- Consent to take part in the study
- Confidentiality, explain how the data will be used
- Interview will last approximately 45-60 minutes
- Audio recorded to ensure interviewer can fully engage in the interview

**Warm up questions:** *Aim\ make participants comfortable*

1. Please introduce yourself?

2. How old are you?

3. What is your education level?

4. What is your job?

5. Are you single or married?

6. How long has it been since the incident (rape)?

**Interview guide questions in individual interviews with women survivors of rape**

1. What problems have you faced in receiving health services since this incident (rape)?

2. What needs in terms of providing care and services have you felt since then? Please explain about it?

3. How could service providers help you? Please explain?

4. What would you like service providers to do for you? Please explain?

5. In general, what do you expect from the health system to improve your situation?

**Additional file 2:** Interview guide during the face-to-face interviews with service providers (midwives, emergency medicine specialists, gynecologists, forensic medicine specialists, reproductive health specialists, infectious disease specialists, psychiatrists, psychologists, general practitioners, social workers and lawyers) for the study conducted to explore the needs related to the health system from the perspective of women survivors of rape and service providers in Isfahan Town, Iran, 2021-2022 (See methods section for further description).

**Introduction:** *Aim, to create appropriate atmosphere*

- Name of the interviewer and affiliation
- Purpose of the study
- Consent to take part in the study
- Confidentiality, explain how the data will be used
- Interview will last approximately 45-60 minutes
- Audio recorded to ensure interviewer can fully engage in the interview

**Warm up questions:** *Aim\ make participants comfortable*

1. Please introduce yourself?

2. How old are you?

3. What is your education level?

4. What is your job?

5. What is your work experience?

**Interview guide questions in individual interviews with service providers**

1. In your opinion, what are the needs of women survivors of rape in terms of providing care and services in the health system? Please explain about it?

2. What can service providers do to meet the needs of these women?

3. In your opinion, what should health policymakers do to improve the status of women survivors of rape?
